# Supplementary material for: Dwarf Pomegranate (Punica granatum L. var. nana): Source of 5-HMF and Bioactive Compounds with Applications in the Protection of Woody Crops
Source: Plants (Basel). 2022 Feb 18;11(4):550. doi: 10.3390/plants11040550 (PMC8879262; doi:10.3390/plants11040550)
Supplement: Supplementary file 1 [file plants-11-00550-s001.zip › plants-1572874-supplementary.pdf]

Dwarf pomegranate (*Punica granatum* L. var. *nana*): source of 5-HMF and bioactive compounds with applications in the protection of woody crops

E. Sánchez-Hernández, L. Buzón-Durán, J.A. Cuchí-Oterino, J. Martín-Gil,
B. Lorenzo-Vidal and P. Martín-Ramos

SUPPLEMENTARY MATERIALS

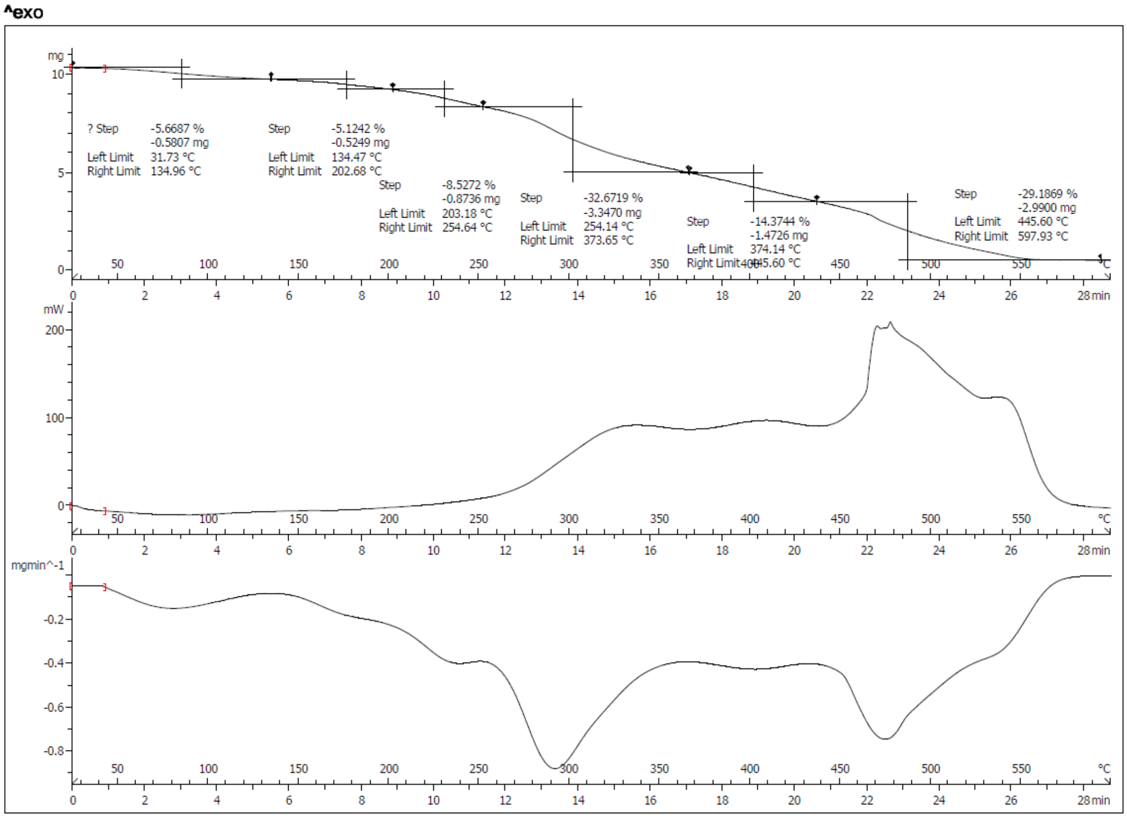

Figure S1. TG, DSC, and DTG curves for *P. granatum* var. *nana* fruits.

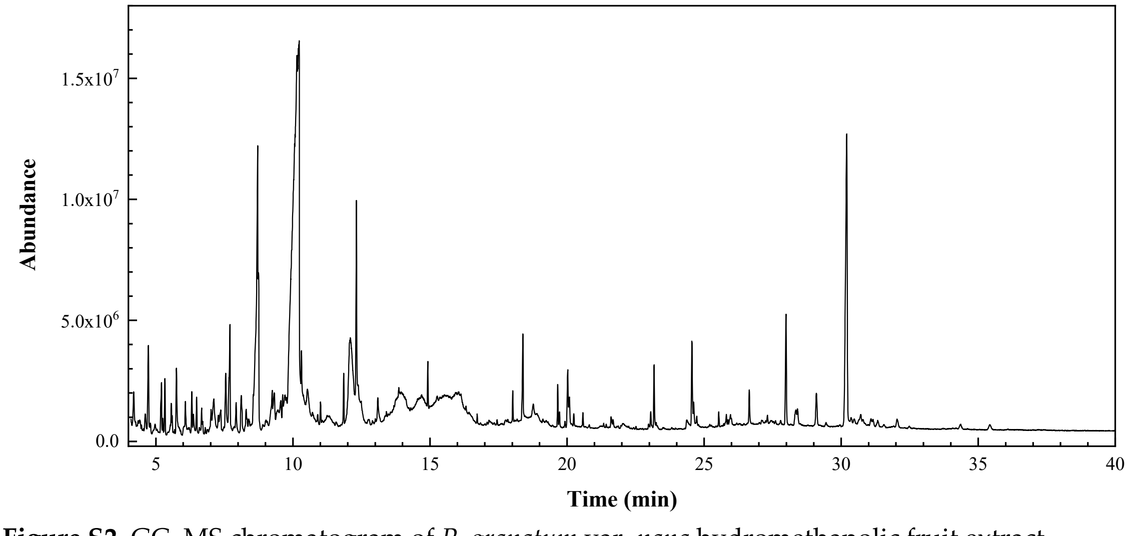

Figure S2. GC−MS chromatogram of *P. granatum* var. *nana* hydromethanolic fruit extract.

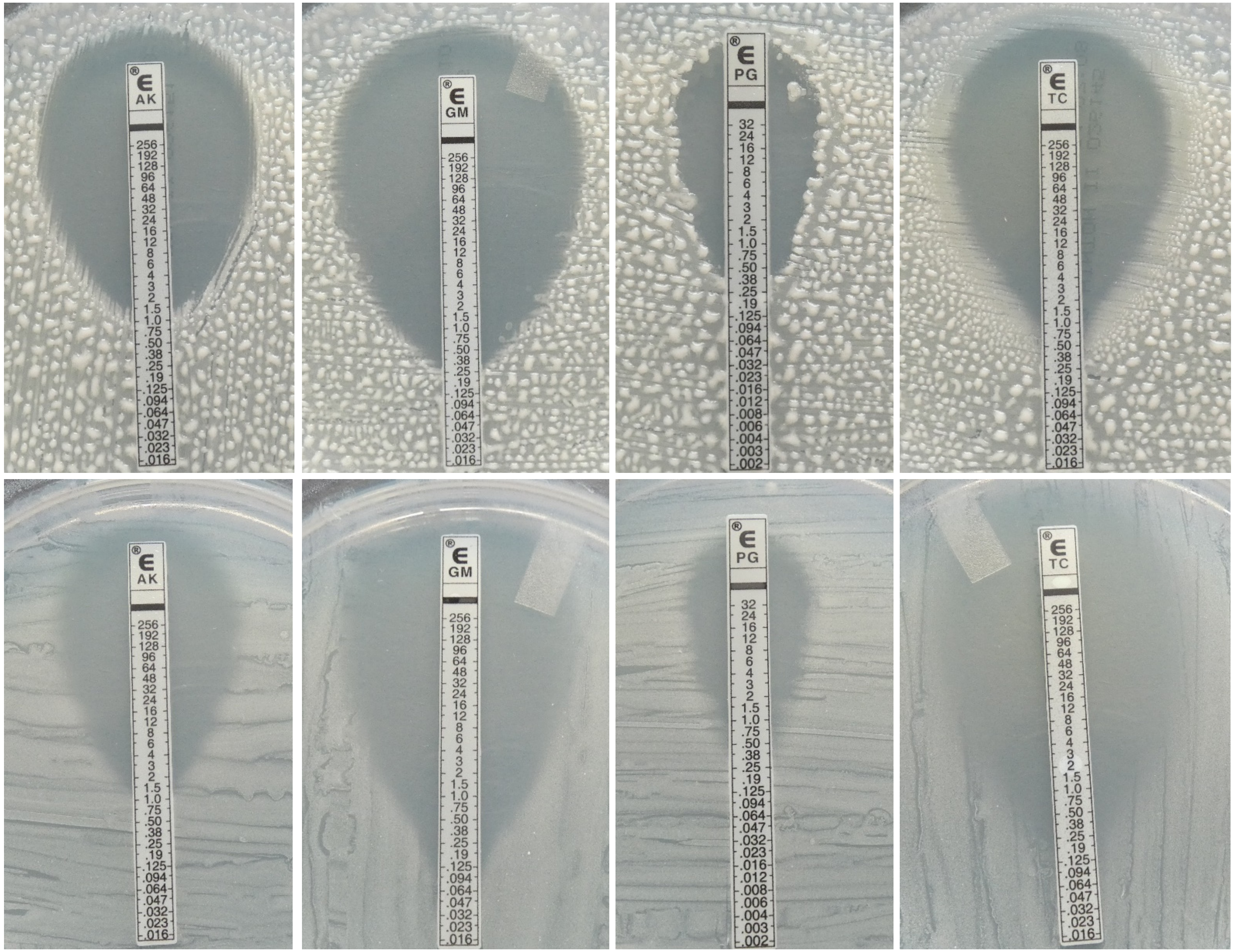

Figure S3. Determination of minimum inhibitory concentrations of conventional antibiotics against *Erwinia amylovora* (*top row*) and *Erwinia vitivora* (*bottom row*) using ETEST® gradient MIC strips. *From left to right*: AK = amikacin, GM = gentamicin, PG = benzylpenicillin, TC = tetracycline.

Table S1. Minimum inhibitory concentrations of conventional antibiotics against *Erwinia amylovora* and *E. vitivora* (expressed in μg·mL^{-1}).

Bacteria	AK	GM	PG	TC
Erwinia amylovora	1.5	0.38	0.5	1.0
Erwinia vitivora	1.5	0.19	2.0	0.5

AK = amikacin, GM = gentamicin, PG = benzylpenicillin, TC = tetracycline

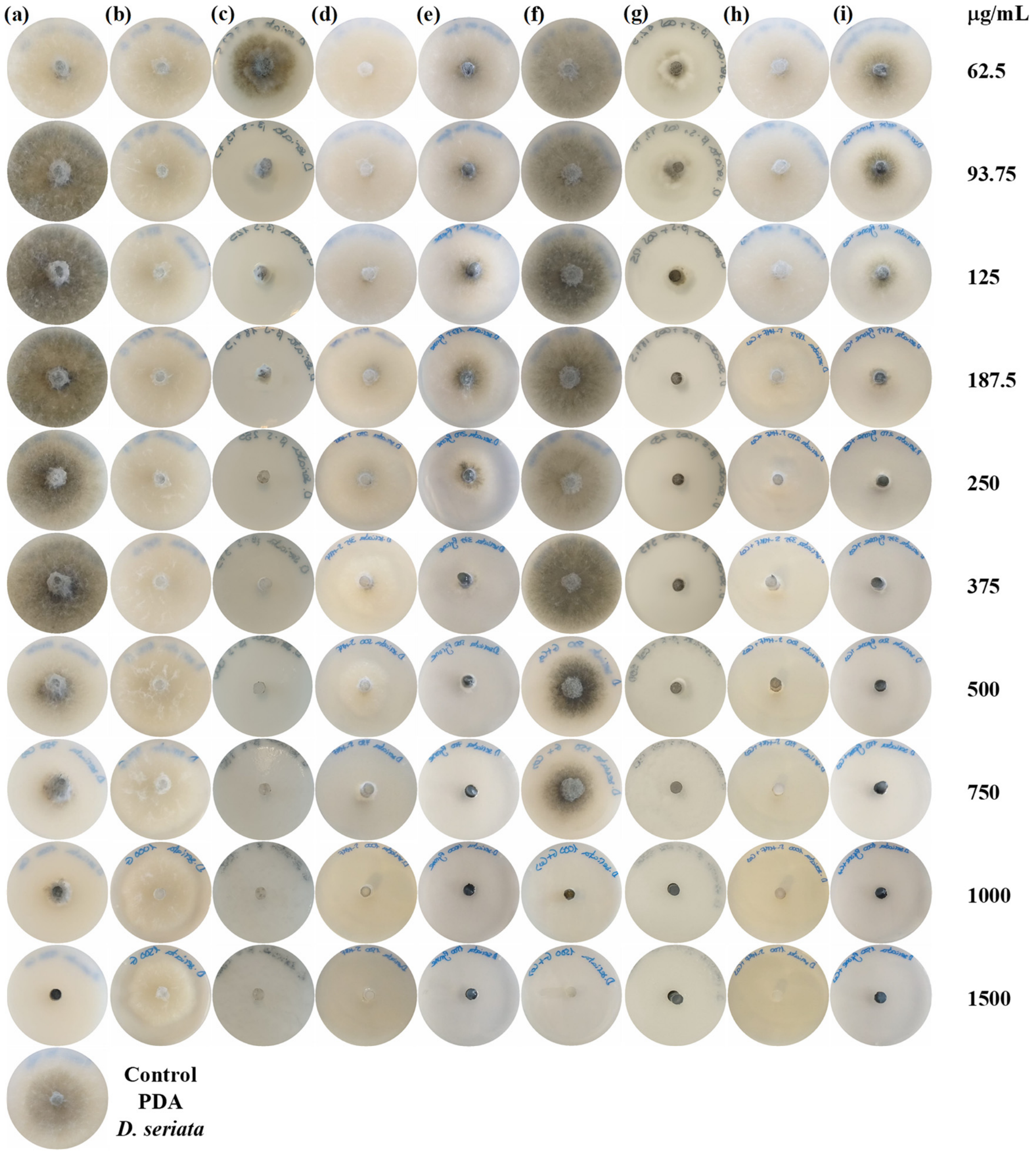

Figure S4. Mycelial growth inhibition of *D. seriata* upon treatment at different concentrations with **(a)** chitosan oligomers, COS; **(b)** *P. granatum* var. *nana* fruits hydromethanolic extract; **(c)** *β*-sitosterol; **(d)** 5-HMF; **(e)** DDPM; **(f)** COS–*P. granatum* conjugate complex; **(g)** COS–*β*-sitosterol conjugate complex; **(h)** COS–5-HMF conjugate complex; and **(i)** COS–DDPM conjugate complex. Only one replicate is shown. The control (PDA-only medium, without any amendments) is shown in the bottom left corner.
